# *Acinetobacter baumannii* neonatal mastitis: a case report

**DOI:** 10.1186/1752-1947-8-318

**Published:** 2014-09-25

**Authors:** Emma L Mohr, Abeba Berhane, John Gregory Zora, Parminder S Suchdev

**Affiliations:** 1 Emory University Pediatrics Residency Program, 49 Jesse Hill Jr. Dr. SE, Atlanta, GA 30303, USA; 2 Emory University School of Medicine, 1648 Pierce Dr. NE, Atlanta, GA 30322, USA; 3 Department of Pediatrics, Emory University, 1405 Clifton Rd, Atlanta, GA 30322, USA

**Keywords:** *Acinetobacter baumannii/haemolyticus*, Febrile infant, Neonatal mastitis

## Abstract

**Introduction:**

Neonatal mastitis is a rare infection. When it does occur, infants younger than 2 months of age are typically affected and the majority of cases are caused by *Staphylococcus aureus.* We present the first reported case of neonatal mastitis caused by *Acinetobacter baumannii*, an unusual organism for this type of infection*.*

**Case presentation:**

A 15-day-old full-term Caucasian male neonate presented to our emergency room following fever at home and was admitted for routine neonatal sepsis evaluation. After admission, he developed purulent drainage from his right nipple, was diagnosed with mastitis, and was started on empiric therapy with clindamycin and cefotaxime with presumed coverage for *S. aureus*. Drainage culture identified pan-susceptible *Acinetobacter baumannii/haemolyticus* and antibiotic therapy was changed to ceftazidime. He was discharged after 5 days of ceftazidime with complete resolution of his symptoms.

**Conclusions:**

This case illustrates the importance of obtaining drainage cultures in mastitis cases because of the possibility of organisms besides *S. aureus* causing infection. *Acinetobacter baumannii* is considered part of the normal human flora and is associated with serious infections in intensive care units. This is the first case report describing *Acinetobacter baumannii* as an etiologic agent of neonatal mastitis and highlights the importance of including unusual organisms in the differential for infectious etiologies for general practitioners.

## Introduction

The most common etiologic agents of neonatal mastitis are *Staphylococcus aureus* and, less commonly, group B Streptococcus, group A Streptococcus, *Escherichia coli*, *Bacteroides* species, *Klebsiella pneumoniae*, and *Enterococcus*[1,2]. A recent chart review determined the prevalence of infecting organisms in 130 patients from breast abscess cultures as follows: methicillin-resistant *S. aureus* (38%), methicillin-susceptible *S. aureus* (35%), coagulase-negative *Staphylococcus* (15%), group B Streptococcus (4%) and *Enterobacter aerogenes* (2%) [3]. Empiric antibiotic coverage for *S. aureus*, tailored for either methicillin-resistant or methicillin-sensitive strains based on the local community prevalence, should be initiated upon diagnosis of mastitis [3]. We present to the best of our knowledge the first case describing *Acinetobacter baumannii* as an etiologic agent of mastitis.

## Case presentation

A 15-day-old full-term Caucasian infant presented to our emergency room with fever (maximum rectal temperature of 38.7°C/101.7°F), decreased urine output, decreased feeding, lethargy and irritability for 1 day. He was born vaginally at 41 weeks and 6 days with no complications. On physical examination, his rectal temperature was 37.6°C and he appeared well. His umbilical stump was not erythematous and had no purulent drainage. He had no rashes or vesicles. His laboratory examination in our emergency department demonstrated an elevated white blood cell count of 15,500, with 76% neutrophils and 13% lymphocytes on the differential. A urine analysis and chest X-ray were reassuring. Blood and urine cultures were obtained. A lumbar puncture was attempted twice, but was unsuccessful. He was admitted to our general pediatrics service on no antibiotics, but with the plan to obtain cerebrospinal fluid (CSF) with another lumbar puncture and initiate antibiotics if he became febrile.

Within a few hours of admission, he developed right breast induration, erythema around his nipple (4cm diameter), and purulent drainage. The wound drainage was cultured. He was empirically treated with clindamycin and cefotaxime, with coverage for methicillin-resistant *S. aureus*, which is prevalent in the area.

His right nipple continued to drain 2 to 3mL of purulent material over the next 24 hours. Because he remained afebrile, was otherwise healthy appearing, and had a source of infection to explain the presenting fever, no lumbar puncture was performed. His blood and urine cultures were negative after 5 days. The right breast drainage, erythema and induration improved significantly within 24 to 36 hours, and completely resolved by day 4 of admission. His right breast wound culture initially revealed Gram-negative rods on Gram stain, and ultimately grew *Acinetobacter baumannii/haemolyticus*. His isolate was susceptible to all the antibiotics tested, including amikacin, cefepime, ceftazidime, ciprofloxacin, gentamicin, levofloxacin, meropenem and tobramycin.

Our pediatric infectious diseases consultant recommended 5 days of treatment with intravenous ceftazidime for mastitis secondary to *Acinetobacter baumannii/haemolyticus* infection. He tolerated this antibiotic regimen with no appreciated adverse effects and continued to breastfeed well during his admission. At discharge, he showed no clinical signs of mastitis, and was not prescribed any oral antibiotics.

## Conclusions

No cases of neonatal mastitis secondary to *Acinetobacter* species infections have been previously documented, so this finding was unexpected. *A. baumannii* is an aerobic, Gram-negative coccobacillus that is considered part of the normal human flora of the skin and mucous membranes of the pharynx, human respiratory secretions, urine and rectum [4]. Of interest, *Acinetobacter* species are the only Gram-negative bacteria that are considered part of the normal flora on human skin, and up to 42.5% of healthy individuals are colonized with *Acinetobacter* species [4]. They also colonize animals, including farm animals, birds, fish, chickens and cats [4]. These bacteria survive on inanimate objects, in dry conditions, in dust, and in moist conditions for several days [4]. The definitive source of *Acinetobacter* species infection in this case of a previously healthy full-term neonate remains unknown, although possible sources include the local environment or contact through the patient’s father’s employment in a veterinary office.

*Acinetobacter* species are most commonly associated with opportunistic infections in war veterans, serious nosocomial infections in intensive care units, and increased resistance to multiple classes of antibiotics. In the pediatric population, most (89%) *Acinetobacter* outbreaks occur in neonatal intensive care units, and bacteremia and meningitis are the most common clinical presentations of infection [5]. However, invasive *Acinetobacter* infections from community settings can also occur, although mainly in warm humid climates [5]. In a case series of Kenyan infants admitted from the community, 10.1% of all positive blood cultures grew *Acinetobacter* species [6].

Skin abscesses in pediatric patients due to *Acinetobacter* have been reported in only two case reports. One described an abscess in the antecubital fossa at the site of a previous intravenous cannula in a neonate born at 26 weeks who previously grew *A. baumannii* from endotracheal tube secretions. This infant appeared well during the initial presentation of the abscess; however, almost 1 week after abscess development, the neonate developed sepsis and blood cultures grew *A. baumannii* and *Enterococcus*. He recovered with antibiotic treatment with amoxicillin-clavulanate and gentamicin [7]. A second case of a superficial abscess that grew *Acinetobacter* was also described in a 24-day-old female infant admitted for birth asphyxia at 2 days of life who subsequently developed an abscess at the site of intravenous scalp vein cannulation. Wound cultures from the abscess grew *Acinetobacter calcoaceticus* var. *anitratus*. This infant developed meningitis and CSF cultures grew the same organisms with the same susceptibilities. She recovered with antibiotic treatment with trimethoprim-sulfamethoxazole [8].

Our patient had negative blood and urine cultures, but we were initially unable to obtain CSF, so no CSF culture was performed. Whether all well-appearing infants with mastitis should receive a lumbar puncture as part of a complete sepsis evaluation is debatable [3,9,10]. Multiple chart reviews have found no positive concordant cultures from urine, blood, or CSF in infants with positive breast cultures, suggesting that there is a low risk for bacteremia in otherwise well-appearing infants with mastitis [2,3,9]. However, lumbar puncture should still be considered for febrile infants <2-months old because of the risk of serious bacterial infection [3].

In conclusion, although most breast abscesses are caused by *S. aureus* and empiric antibiotic coverage should include this organism, less common infections also need to be considered. This case highlights the importance of obtaining cultures from breast abscesses because of the possibility of an uncommon infecting organism and provides a brief introduction to *Acinetobacter* species infections in the pediatric population.

## Consent

Written informed consent was obtained from the patient’s parent for publication of this case report. A copy of the written consent is available for review by the Editor-in-Chief of this journal.

## Abbreviations

CSF: Cerebrospinal fluid.

## Competing interests

The authors declare that they have no competing interests.

## Authors’ contributions

EM, AB, JZ and PS participated in conception and design of this report and were involved in drafting and revising the manuscript. EM participated in final revisions of the manuscript. All authors read and approved the final manuscript.
